# Insight into the Structure and Dynamics of Polymers by Neutron Scattering Combined with Atomistic Molecular Dynamics Simulations

**DOI:** 10.3390/polym12123067

**Published:** 2020-12-21

**Authors:** Arantxa Arbe, Fernando Alvarez, Juan Colmenero

**Affiliations:** 1 Centro de Física de Materiales (CSIC, UPV/EHU) and Materials Physics Center MPC, Paseo Manuel de Lardizabal 5, E-20018 San Sebastián, Spain; a.arbe@ehu.eus (A.A.); fernando.alvarez@ehu.eus (F.A.); 2 Departamento de Polímeros y Materiales Avanzados, Física, Química y Tecnología (UPV/EHU), Apartado 1072, E-20080 San Sebastián, Spain; 3 Donostia International Physics Center (DIPC), Paseo Manuel de Lardizabal 4, E-20018 San Sebastián, Spain

**Keywords:** structure of polymers, dynamics of polymers, neutron scattering, fully atomistic molecular dynamics simulations

## Abstract

Combining neutron scattering and fully atomistic molecular dynamics simulations allows unraveling structural and dynamical features of polymer melts at different length scales, mainly in the intermolecular and monomeric range. Here we present the methodology developed by us and the results of its application during the last years in a variety of polymers. This methodology is based on two pillars: (i) both techniques cover approximately the same length and time scales and (ii) the classical van Hove formalism allows easily calculating the magnitudes measured by neutron scattering from the simulated atomic trajectories. By direct comparison with experimental results, the simulated cell is validated. Thereafter, the information of the simulations can be exploited, calculating magnitudes that are experimentally inaccessible or extending the parameters range beyond the experimental capabilities. We show how detailed microscopic insight on structural features and dynamical processes of various kinds has been gained in polymeric systems with different degrees of complexity, and how intriguing questions as the collective behavior at intermediate length scales have been faced.

## 1. Introduction

**Figure 1:**
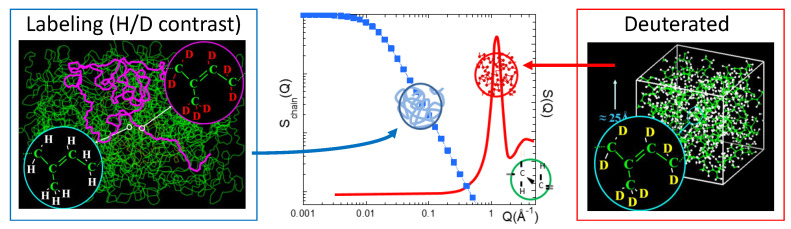
Cartoon illustrating the relevant structural features of polymers as function of the length scale of observation and magnitudes accessed in neutron diffraction experiments: the single chain structure factor on labelled samples at low scattering angles (**left**) and the structure factor on fully deuterated samples (**right**).

The structural units of polymers are macromolecules. For this reason, their structural and dynamical properties strongly depend on a hierarchy of length and time scales. Considering structural aspects, nowadays it is well established the random coil conformation of linear polymer chains in the melt and the glassy state. This conformation was envisioned by Flory in the 50’s [1]. Reducing the length scale of observation, the local arrangements of the atoms leading to short-range order give rise to broad maxima in the diffraction patterns. Intermolecular correlations are revealed by the ‘amorphous halo’ in the range of scattering vector Q≈ 1 ${Å}^{-1}$ (see Figure 1). Associated to the structural complexity, the dynamical processes in polymers also depend on the length scale considered. At large length scales polymer melts display unique properties driven by the chain-like character and the atomic decoration of the chain plays a secondary role. At such large scales, the relevant dynamic processes (chain diffusion, reptation and Rouse dynamics) exhibit characteristic times that depend on the macromolecular size and can be very long. At intermolecular length scales the so-called α-relaxation is the main dynamical process [2]. The characteristic time of this relaxation—the structural relaxation time—raises enormously when the temperature decreases towards the glass transition temperature $T_{g}$. The behavior of polymers at these length scales is dominated by the universal features of glass-forming systems. At even smaller length scales ≈ 1 Å the most relevant motions are usually localized and activated and persist in the glassy state. The structural disorder leads to the emergence of broad barrier distributions for such processes. This kind of dynamics comprises, among others, tunneling processes [3], secondary relaxations like the Johari–Goldstein or β-relaxation [4] and vibrations including the Boson peak [5].

Unveiling the structural and dynamical properties of polymeric systems at the different levels and identifying their interplay is a great challenge. In addition, the consideration of different ingredients that increase the complexity of the system (e.g., chemically more complex monomeric units, different macromolecular architectures, polymer blends, nanocomposites, …) can lead to profound modifications of the features identified and characterized in chemically simple linear homopolymer melts. Even novel properties can emerge as consequence of complexity. In the following we present the strategy developed by us in order to tackle these questions [6]. It rests on the synergetic combination of two techniques: neutron scattering (NS) and fully atomistic molecular dynamics (MD) simulations. After setting the methodological aspects, we review the main achievements we have obtained by applying it to the investigation of structural and dynamical features of ‘simple’ linear homopolymers. This is followed by a section where more complex and intricate systems and situations are addressed. Finally, we present our conclusions and considerations about the extension of this kind of strategy to tackle problems in systems displaying other kinds of complexity.

## 2. Grounds of the Strategy

Neutrons interact with the atomic nuclei [7,8]. The involved interactions are important only at very short distances ($|\vec{r}|\;\approx \;10^{-14}$ m) and can be approximately described by

(1) $$V\left(\vec{r}\right)=-\frac{2\pi \hbar^{2}}{m}b_{\alpha }\delta \left(\vec{r}\right)$$

with *m* the mass of the neutron and $b_{\alpha }$ the scattering length. The scattering length depends on the isotope α (α: H, D, C, O, …) and also on the relative orientation of the neutron-nuclear spin pairs. The scattering length can be positive, negative or complex. Table 1 shows its mean value, $\bar{b_{\alpha }}$, for the isotopes commonly present in soft materials.

**Figure 2:**
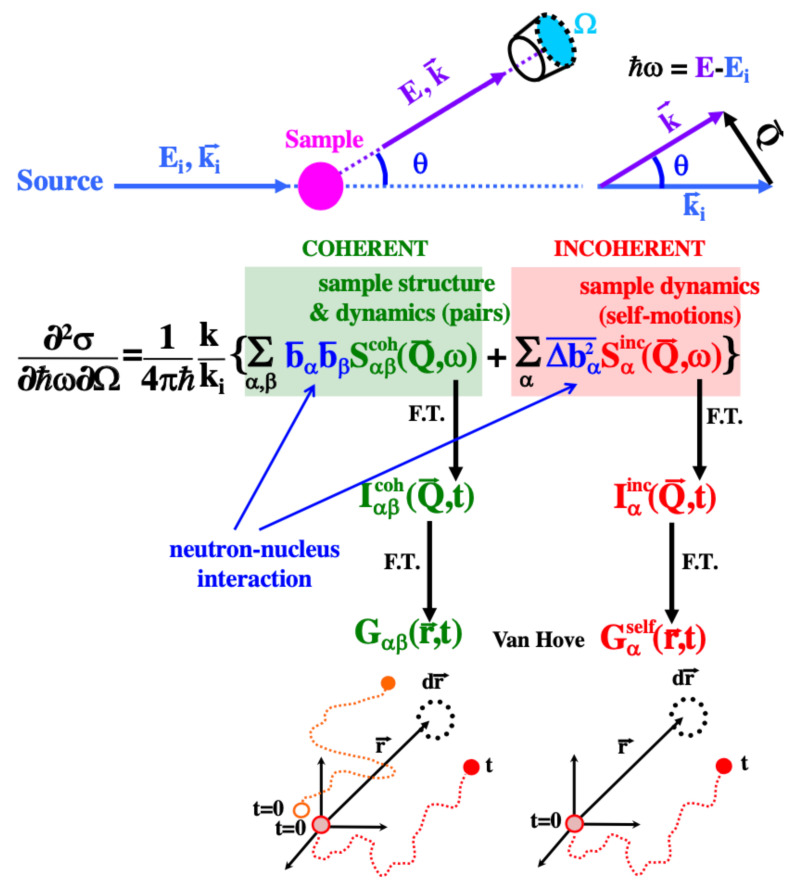
Illustration of the magnitudes in a scattering experiment and scheme of the functions involved in the different domians in the van Hove formalism of neutron scattering.

The magnitude measured in NS experiments is the cross section (see Figure 2). The number of neutrons scattered into a solid angle comprised between Ω and Ω+dΩ and which have changed their energy by an amount ℏω, with respect to the total number of incident neutrons [7], is the double differential scattering cross section $\partial^{2}\sigma /\partial \Omega \partial \hbar \omega$. The difference between the wavevectors of the scattered ($\vec{k}$) and incident ($\vec{k_{i}}$) neutron determines the scattering vector $\vec{Q}$ (equivalently, the momentum transfer $\hbar \vec{Q}$). In an elastic experiment (ℏω=0), since $k\;=\;k_{i}$, the modulus of this vector is given by $Q\;=\;2k_{i}\sin\left(\theta /2\right)$. Here θ is the scattering angle. This expression can be considered as a good approximation for small energy transfers—as usually involved in the so-called quasielastic experiments, where small energy transfers are considered. The interpretation of $\partial^{2}\sigma /\partial \Omega \partial \hbar \omega$ is straightforward in terms of the so-called van Hove correlation functions, as shown in the scheme of Figure 2. In this scheme, indexes α and β run over all the possible kinds of isotopes (α,β: H, D, C, O, N, …) in the sample. $\partial^{2}\sigma /\partial \Omega \partial \hbar \omega$ contains a coherent and an incoherent contribution. The latter has its origin in the random distribution of the deviations of the scattering lengths from their mean value, i.e., it is defined as $\bar{\Delta b_{\alpha }^{2}}=\bar{b_{\alpha }^{2}}-{\bar{b_{\alpha }}}^{2}$. The $\vec{Q}$- and ω-dependencies of coherent and incoherent contributions are determined by the corresponding scattering functions. They are respectively $S_{\alpha \beta }^{coh}\left(\vec{Q},\omega \right)$ that involves nuclei of kinds α and β, and $S_{\alpha }^{inc}\left(\vec{Q},\omega \right)$ that involves nuclei of kind α. The scattering functions are related, via Fourier transformation, with the intermediate scattering functions [$I_{\alpha \beta }^{coh}\left(\vec{Q},t\right)$ and $I_{\alpha }^{inc}\left(\vec{Q},t\right)$] and the van Hove correlation functions [$G_{\alpha \beta }\left(\vec{r},t\right)$ and its self-part $G_{\alpha }^{self}\left(\vec{r},t\right)$]. We note that the functions $I_{\alpha \beta }^{coh}\left(\vec{Q},t\right)$ and $I_{\alpha }^{inc}\left(\vec{Q},t\right)$ are also denoted as $S_{\alpha \beta }^{coh}\left(\vec{Q},t\right)$ and $S_{\alpha }^{inc}\left(\vec{Q},t\right)$, respectively. The label ‘inc’ is sometimes substituted by ‘self’ or simply ‘s’ denoting ‘self’. In the classical limit, $G_{\alpha \beta }\left(\vec{r},t\right)$ is expressed as:

(2) $$G_{\alpha \beta }\left(\vec{r},t\right)=\langle \frac{1}{N}\sum_{i\alpha ,j\beta }^{N_{\alpha },N_{\beta }}\delta \left\{\vec{r}-\left[{\vec{r}}_{i\alpha }\left(t\right)-{\vec{r}}_{j\beta }\left(0\right)\right]\right\}\rangle .$$

Here ${\vec{r}}_{i\alpha }\left(t\right)$ [${\vec{r}}_{j\beta }\left(0\right)$] is the position vector of the *i*th atom of kind α [*j*th atom of kind β] at time = *t* [time = 0]. All the different atoms of kinds α and β [$N_{\alpha }$ ($N_{\beta }$): total number of atoms of kind α (β); $N\;=\;\sum_{\alpha }N_{\alpha }$] are included in the sum. Thus, $G_{\alpha \beta }\left(\vec{r},t\right)d\vec{r}$ is the probability that any particle of kind α is in the volume $d\vec{r}$ at position $\vec{r}$ at time *t*, if a particle of kind β is at the origin at time *t* = 0. In the static case $G_{\alpha \beta }\left(\vec{r},t=0\right)=\delta_{\alpha \beta }\left(\vec{r}\right)+g_{\alpha \beta }\left(\vec{r}\right)$, where $g_{\alpha \beta }\left(\vec{r}\right)$ is the static pair distribution function. Thus, neutrons provide structural information and, through the time-dependent correlation functions, they also reveal dynamical collective properties. On the other hand, the self-part of the van Hove correlation function $G_{\alpha }^{self}\left(\vec{r},t\right)$ is obtained by restricting the correlations considered in Equation (2) to those relating the positions of a single particle of kind α at different times:

(3) $$G_{\alpha }^{self}\left(\vec{r},t\right)=\langle \frac{1}{N}\sum_{i\alpha }^{N_{\alpha }}\delta \left\{\vec{r}-\left[{\vec{r}}_{i\alpha }\left(t\right)-{\vec{r}}_{i\alpha }\left(0\right)\right]\right\}\rangle .$$

$G_{\alpha }^{self}\left(\vec{r},t\right)$ is the Fourier transform of $I_{\alpha }^{inc}\left(\vec{Q},t\right)$ in space: Incoherent scattering relates to single particle motions, i.e., informs about the motions of an individual nucleus (or ‘self’ motions).

The main equation in Figure 2 shows that the weights of the coherent and incoherent contributions to the scattered intensity are determined by the scattering lengths of the isotopes involved. We also define the so-called scattering cross sections of the sample, $\sigma_{coh}$ and $\sigma_{inc}$, in terms of the scattering cross sections of its individual nuclei $\sigma_{coh(inc)}^{i}$: coherent (incoherent) scattering cross section of nucleus i (see Table 1). The definition of the sample cross section is $\sigma_{coh(inc)}=\sum \sigma_{coh(inc)}^{i}/N$, while the atomic cross sections are expressed as $\sigma_{coh}^{i}=4\pi {\bar{b_{i}}}^{2}$, $\sigma_{inc}^{i}=4\pi \bar{\Delta b_{i}^{2}}$ [7,9]. From Table 1 it is follows that:

- Due to the large value of $\bar{\Delta b_{H}^{2}}$ (and, thus, of $\sigma_{inc}^{H}$), in H-containing systems $\sigma_{inc}>>\sigma_{coh}$ and the signal is dominated by the incoherent scattering from hydrogens. It thus reveals their self-motions
- If H is substituted by D, this incoherent contribution is drastically reduced. Then, we obtain differently weighted coherent contributions
- Since $\sigma_{coh}>>\sigma_{inc}$ in a fully deuterated sample, the intensity scattered is mainly coherent. Given that $\bar{b_{D}}\approx \bar{b_{C}}$, all pair correlations are almost equally weighted

Neutrons also provide an elegant—and unique—way to experimentally access another kind of correlation functions. Thanks to the large difference between $\bar{b_{H}}$ and $\bar{b_{D}}$, they can address the dynamic structure factor of isotopically labeled molecular groups or macromolecules in experiments sensitive to energy changes. In diffraction experiments, its static counterpart, the corresponding form factor, is accessed. We recall that the dynamic structure factor of a labeled ’object’ containing *z* scattering points is defined as $S_{{}^{\prime }object^{\prime }}\left(\vec{Q},t\right)\;=\;z^{-2}\sum_{i}\sum_{j}\langle \exp\left(-i\vec{Q}{\vec{r}}_{ij}\left(t\right)\right]\rangle$. Here the sums extend to all the scattering points in the ’object’, and ${\vec{r}}_{ij}\left(t\right)$ is the vector joining two scattering points. The static and dynamic structure factors are revealed by the cross section measured on a mixture of protonated and deuterated ‘objects’ at low scattering angles (see Figure 1 for the example of a polymer melt). The scattered intensity is then weighted by the scattering contrast factor $\Delta \rho^{2}\;\propto \;{\left(\bar{b_{H}}-\bar{b_{D}}\right)}^{2}$. This unique opportunity offered by neutrons made it possible to experimentally prove [10,11] the random coil conformation of macromolecules in the bulk. Those pioneering experiments were realized in the 70’s, as soon as the first small angle neutron scattering diffractometers were available, and are one of the greatest historical milestones in polymer physics. Only then was Flory awarded with the Nobel Prize. Later, dynamic measurements have provided microscopic evidence for the Rouse-like [12] dynamics in unentangled chains [13] and the reptation mechanism for entangled chain dynamics [14]. This was predicted by Doi, Edward and deGennes [15,16] in the 80’s. Thus, with NS we can also ‘see’ how ensembles of atoms (like those in a macromolecule) look like and move!

To perform neutron scattering experiments, large facilities including a neutron source and dedicated instrumentation (see, e.g., [17]) are needed. Currently, the effective (Q,t) region covered by NS techniques is roughly 0.01 < *Q* < 2 ${Å}^{-1}$ and 0.1 ps < *t* < 100 ns. Neutrons have been used to investigate at microscopic level *all* the different dynamical processes taking place in linear homopolymer materials [13] over the past decades.

Neutron scattering experiments provide thus a big deal of information and the magnitudes measured have a clear physical meaning. However, their analysis and interpretation is sometimes very difficult, given the complexity of soft materials. In addition to the intrinsic problem of properly modeling the structural and dynamical complexity, several problems arise from the limitation of NS techniques, namely:

1. NS accesses correlation functions in the reciprocal space ($\vec{Q}$), never in real space
2. NSE does not distinguish the signals of different atoms, if they are of the same isotopic species (e.g., main-chain hydrogens vs side-group hydrogens)
3. Self-motions of C and O are not accessible ($\bar{\Delta b_{C}^{2}}$ = $\bar{\Delta b_{O}^{2}}$ = 0)
4. With exception of the neutron spin echo (NSE) technique (F. Mezei, 1972 [18]), that directly accesses the intermediate scattering functions, experiments are performed in the frequency domain and the results are affected by the instrumental resolution through convolution
5. Spectrometers cover relatively narrow dynamic windows and usually several instruments have to be combined
6. Though polarization analysis (PA) allows separation of coherent and incoherent contributions, in the practice this is currently available only for diffraction experiments (structural information)

Some of these limitations (4–6) might be overcome with the development of the neutron sources and instrumentation, in particular with the spallation sources of new generation. In fact, recent progresses in instrumentation have allowed PA also in quasielastic neutron scattering experiments, though not yet in a routinely way [19]. However, points (1–3) are inherent to the scattering processes.

Computer simulations can be of utmost help for the interpretation of NS results. In the following we introduce these techniques and the way their combination with NS allows a full exploitation of their capabilities.

**Figure 3:**
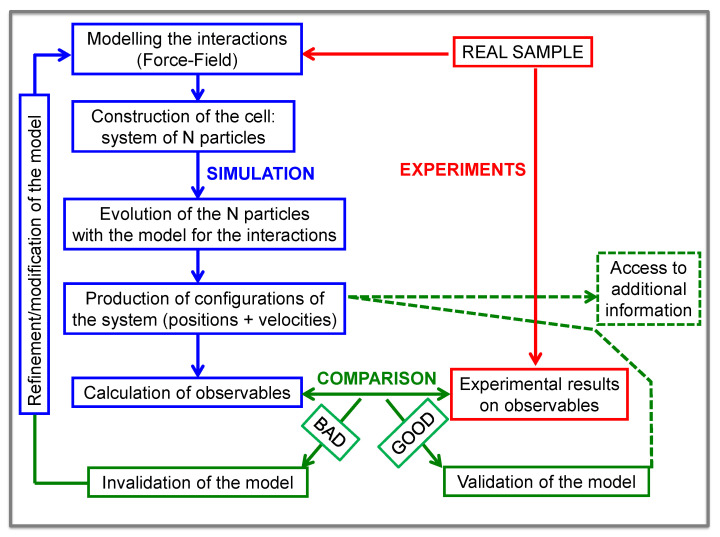
Block-diagram of the strategy followed for combining NS and MD-simulations.

It is considered that computer simulations in general—i.e., ab-initio methods, classical molecular dynamics simulations, Monte Carlo methods, etc—are in between theoretical approaches and experimental tools. We could distinguish two points of view when doing simulations: (i) that of a theoretician and (ii) that of an experimentalist. In the first case, the goal is to capture the essence of a given problem and use the simulated system to check theories and theoretical concepts. In this framework, no direct connection with real systems is usually invoked. In the case of the point of view of an experimentalist, the aim is to mimic a real system as faithfully as possible. Then, the best choice should be, in principle, fully atomistic simulations. The thorough validation of the simulated system by comparison with experimental results is crucial in such a case. Once the validation is confirmed—at least for the particular problem in the focus—the simulations can be exploited for e.g., calculating magnitudes that are not experimentally accessible (see Figure 3). Extending the parameters range beyond the experimental capabilities is another opportunity. This feedback involving experiments and simulations allows to understand a given problem and check theories as well.

A classical MD-simulation is just the solution of the classical equations of motion, i.e., Newton’s equations, for a set of *N* particles. The three main elements are (i) to define the system. This involves the construction of the simulated cell at the desired conditions of temperature, pressure, etc.; (ii) to define the interactions between the particles. This consists of selecting the potential energy or ’force-field’, from which forces acting on the particles can be calculated; and (iii) to integrate the equations of motion by a suitable computer algorithm. Figure 3 (blue boxes) shows a typical block-diagram for an atomistic simulation. In such a simulation the particles are atoms. In the first simulation done on a simple liquid ’Argon’ [20] (1954), the number of atoms was 864 in a cubic cell of size 35Å. The force-field was a simple Lennard-Jones potential. The simulation time was only a few ps and periodic boundary conditions were used. In that first work, noteworthy, some of the magnitudes measured by NS were computed, and some comparison (’validation’) with some available NS data was performed. In this sense, that pioneering work could be considered as the first example of the strategy here developed, applied to a very simple liquid. For a polymer melt (a dense ’liquid’ of macromolecules), some specific ingredients have to be additionally considered in order to perform a classical MD-simulation.

First, the big size of macromolecules has to be considered in order to define the construction of the simulated cell. The conformation of a macromolecule in the melt follows more or less the Gaussian statistics. As an example to estimate the required dimensions of a typical simulation cell, we may consider the simplest macromolecule, polyethylene (PE). For this polymer, the ratio between the average squared end-to-end distance $\langle R_{ee}^{2}\rangle$ and the molecular mass, *M*, is given by $\langle R_{ee}^{2}\rangle /M\;\approx$ 1.25$\;{Å}^{2}$g${}^{-1}$mol [21]. For a short chain of 40 monomers (*M* = 1120 g mol${}^{-1}$) this gives $\sqrt{\langle R_{ee}^{2}\rangle }\;\approx \;37\;Å$. Thus, the side of the cubic cell must be at least L≥40Å. A number of atoms N> 6500 would be required, if we consider the density of PE (0.784 g/cm${}^{3}$ at 413 K). This implies that it is really difficult to construct simulation cells for polymer melts that contain the whole macromolecules, even for modest molecular weights. Usually, a ’folding’ procedure involving replicas of the chain and periodic boundary conditions is used. One of them is the so-called amorphous cell protocol proposed many years ago by Suter and Theodorou [22,23]. However, this type of methods limits the capabilities of atomistic MD-simulations for investigating large-scale dynamics even of not very large macromolecules.

Regarding the force-field, the potential energy of an ensemble of atoms belonging to macromolecules can be described as a sum of contributions due to all bond, valence bend and dihedral interactions, and non-bonded interactions. The latter are similar to those used for other organic molecular liquids and glasses. Therefore, the main difference is the diversity of intramolecular contributions associated to the complex macromolecular microstructure. Such a complexity usually makes difficult the actual equilibration of the simulated cell, in particular for large macromolecules, and specially close to glass-transition of the polymer. Different models are being developed to overcome these problems. In particular, those based on the *end-bridging* and *double-bridging* Monte Carlo algorithms (see, e.g., [24,25]). Despite these difficulties, different force fields are currently available for polymers, which yield quite reasonable results—within the limits of applicability commented above. We can cite e.g., the current versions of AMBER [26], COMPASS [27] CHARMM [28], GROMOS [29], OPLS-AA [30], GAFF [31] and DREIDING [32] force fields.

As for the computer algorithm, there are no qualitative differences with respect to MD-simulations of other non-polymeric liquids and glass-forming systems [33]. Different software packages are available—either commercial as for instance *Materials Studio* (Accelrys${}^{R}$, now BIOVIA${}^{TM}$) [34] or of free access as e.g., *Gromacs* [35], *DL POLY* [36], *LAMMPS* [37], *NAMD* [38] or *OCTA* [39]. They allow performing fully atomistic MD-simulations on polymer systems by using different force-fields.

Last, it is worth considering how the simulation time compares with the characteristic time scale of the different dynamic processes in polymer melts. For example, at 100 K above its glass-transition temperature, the time scale of vibrations in a polymer melt would be about 0.1 ps, for methyl-group rotations of the order of 1 ps and for segmental (α-) relaxation can be in the range 1–10 ns. Conversely, large-scale chain relaxation—as for instance the end-to-end relaxation–, involves characteristic times that depend on the molecular mass of the macromolecular structural units (i.e., on the number of monomers $N_{mon}$). For non-entangled polymers, this characteristic time scales with $N_{mon}^{2}$, while for well entangled polymers, with $N_{mon}^{3}$ [15]. Taking e.g., $N_{mon}\;∼\;100$, the characteristic time would be about 10 μs. The standard computing capabilities involve simulation times of the order of 100 ns for simulation cells of about 15,000 atoms. Thus, the characteristic times for large-scale chain relaxation are beyond these capabilities. These problems together with those above mentioned related to the equilibration of the simulation cells, imply that fully atomistic MD-simulations (even united atom simulations) of well-entangled polymer systems are really challenging (see, e.g., [40,41]). For this purpose, MD-simulations based on coarse-grained models are more suitable. There, a blob or bead represents several monomers—as for instance those forming a Kuhn segment, and, in principle, larger macromolecules can be simulated in this way. However, if the level of coarse-graining is increased, other problems can emerge, as e.g., unphysical bond crossing due to the softening of the coarse potentials. In the works described in this review, polymer systems well below the entanglement regime are always considered. Further information about MD-simulations in polymers can be found in the general references [42,43].

Now we address the combination of the two methods—neutron scattering and fully atomistic MD-simulations—in the strategy described here. it is clear that NS techniques are the right tools to validate fully atomistic MD-simulations of diverse systems like liquids and soft materials, and in particular in polymer melts. First of all, these methods cover the relevant length and time scales. In addition, the classical van Hove formalism allows easily calculating the magnitudes measured by NS from the simulated atomic trajectories. Different correlation functions can be experimentally addressed (in particular, thanks to deuterium labelling) and used for exhaustive check of the reliability of the simulated cell. We note that X-ray experiments can also be used as a reference for validation. However, the energy of X-rays is too high to resolve the small energy changes associated to the relaxational processes in polymers, and therefore this kind of studies are restricted to diffraction experiments (ℏω = 0). Therefore, they only give structural information. In addition, since X-photons interact with the electron cloud, they are mostly sensitive to C and O atoms. Labelling techniques are no longer applicable and only one partial structure factor is accessed. As can be appreciated, neutrons provide much more demanding tests for the validation of the simulation.

Neutron scattering techniques on one hand and molecular dynamics simulations on the other hand have been extensively employed to study polymer structural and dynamical properties in the past. However, the synergetic combination we propose here has only been rarely applied. We can also mention some representative works by other authors [44,45,46,47,48,49,50,51,52,53,54,55]. We may emphasize here again that the exploitation of fully atomistic MD-simulations is trustworthy, of course, within the limits above discussed, and for the particular problem they can be validated by direct comparison with experimental results. In the following section we illustrate how this strategy has allowed us disentangling structural and dynamical properties of glass-forming polymers spanning over different length scales.

## 3. Results on ‘Simple’ Linear Homopolymers

### 3.1. Structural Properties

The local structure of many polymer materials is still poorly known. This structure emerges in a diffraction experiment in the *Q*-range $Q\;>\;0.5\;{Å}^{-1}$. There, the results show two or more broad peaks, sometimes with shoulders. Taking into account their temperature dependence and from qualitative arguments, the peaks in the range between 1 and 1.5 ${Å}^{-1}$ approx. are assumed to have an intermolecular character. Conversely, those appearing at higher *Q*-values are intra-molecular in nature [56] (see e.g., Figure 1). However, in many cases, the particular atomic or molecular correlations behind these peaks, i.e., the actual short-range order in the real space, are not known.

**Figure 4:**
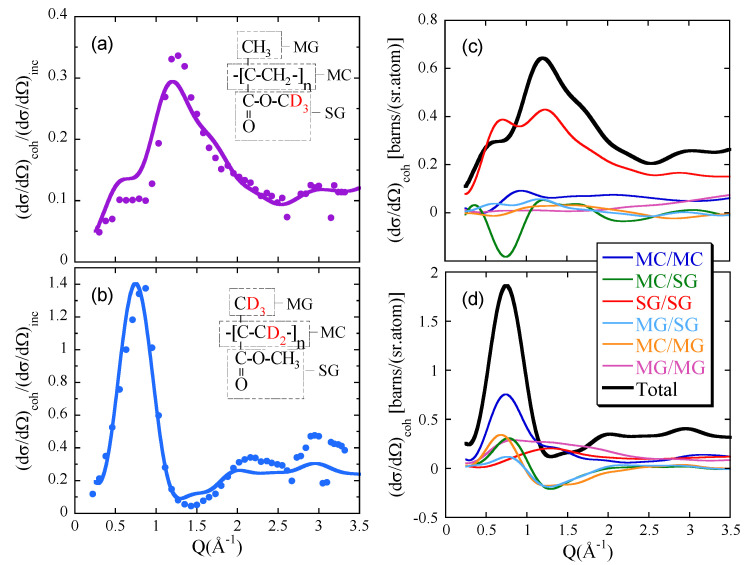
Ratio between coherent and incoherent differential cross sections measured with diffraction with PA (points) and calculated from the MD-simulations (lines) on PMMA with deuterated ester methyl group (PMMA-d3e) (**a**) and PMMA with deuterated α-methyl group and main chain (PMMA-d5) (**b**). The respective coherent cross sections calculated from the simulations with the different molecular group correlations, properly weighted by the corresponding neutron scattering lengths, are shown in panels (**c**,**d**). Adapted with permission from [57]. Copyright (2006) American Chemical Society.

X-ray diffraction delivering only one partial structure factor has been traditionally used for investigating the local structure of polymer systems. Neutron diffraction combined with selective deuteration allows to access different partial structure factors. They include the actual (total) structure factor S(Q). This is obtained in the case of fully deuterated samples. In S(Q) all the atoms are equally weighted (see Figure 2 and Table 1). Experimentally, there is a problem for obtaining the coherent contribution to the diffraction patterns, in particular in protonated or partially protonated samples: the high level of incoherent scattering from the hydrogen atoms. This difficulty is overcome by neutron diffraction with PA, since it nicely allows separating coherent and incoherent contributions. We will present the outcome of the study presented in Ref. [57] on syndiotactic poly(methyl methacrylate) (PMMA, Plexiglass) as a representative example of the potential of the synergetic combination here proposed for structural investigations. Figure 4a,b show two different partial structure factors corresponding to different deuteration levels of PMMA. Some information about the particular atomic correlations behind these peaks can be deduced from a comparative analysis of these partial structure factors. However, MD-simulations on a realistic sample allow going beyond this qualitative analysis. A first step has to be given: to validate the cell ensuring that it reproduces well the experiments. Figure 4a,b show the direct comparison of both kinds of results without any adjustment. The agreement is highly satisfactory. Now the simulations can be exploited to address the short-range structural features in PMMA through the information provided by the partial structure factors. For example, we can easily unveil the different correlations contributing to the structure factors. To gain deep insight into the structure, three molecular substructures can be defined, in terms of which the simulation results may be grouped [57]: the main chain (MC), the α-methyl group (MG), and the ester side group (SG). Figure 4c,d display the resulting decomposition of the partial structure factors shown in panels (a) and (b). The separate correlations can now be analyzed in depth. For example, a characteristic MC/MC distance of $d=2\pi /Q_{max}=8.6\;Å$ could be deduced in the Bragg approximation from the position of the MC/MC main peak $Q_{max}\approx 0.73\;{Å}^{-1}$. Also, a strong anticorrelation of MCs and SGs suggests a precursor effect of the nano-phase separation between the main chains and the side groups observed for methacrylates with larger alkyl side-groups [58].

Furthermore, directly accessing the real space (impossible with NS techniques), the radial probability distribution function corresponding to different key atoms could be inspected [57]. Such analysis pointed to a strong short-range order. In addition, the analysis of the sequence of intra-chain peaks indicated a persistent all-trans structure, in agreement with a locally stiff chain as indicated by the high values reported for the characteristic ratio $C_{\infty }$ [59]. We could also see that intra-chain correlations contribute as well at rather large distances. The analysis of the radial distribution of the ester methyl group carbon suggested some interdigitation of the side groups which again is in agreement with the nano-phase separation precursor effect previously mentioned. We will come back later to this interesting problem.

The strategy here illustrated for PMMA was also applied by us to disentangle the short-range order in other polymers as, for instance: polyisoprene (PI) [60,61,62], polystyrene (PS) [62,63], polybutadiene (PB) [64] or polyisobutylene (PIB) [65].

### 3.2. Dynamical Behavior

#### 3.2.1. Localized Motions

Among the different dynamical processes that take place in polymers, methyl group rotation is perhaps the simplest one, since all the relevant interactions on the methyl group can be condensed in an effective mean-field one-dimensional potential. By means of neutron scattering it is possible to access experimentally the different manifestations of this process, namely quantum tunnelling, classical hopping as well as its fingerprint in the vibrational density of states. The results obtained in many chemically and structurally different polymers [66] can be consistently described in terms of the Rotation Rate Distribution Model (RRDM), which was first proposed in 1994 [67]. This model introduces a distribution of potential barriers for methyl group rotation, which is associated to the disorder present in any structural glass. Interestingly enough, this model predicts, instead of inelastic peaks, an apparent quasielastic broadening for the tunnelling spectrum. This was experimentally found in poly(vinyl acetate) (PVAc) in 1998 [3]. Later, MD-simulations provided further support to the RRDM and allowed extracting interesting information about this process, as we are detailing in the following. Molecular dynamics simulations on PI carried ot at very low temperature [68] were first validated through the comparison of the density of states for methyl group torsional librations, as calculated from the time evolution of the dihedral angles, with previous inelastic neutron scattering results. The librational peak showed a broad feature reflecting a distribution of potential barriers. This distribution was quantified in the framework of the threefold approximation—which was corroborated by the simulations. Then, the capabilities of the computational method were further exploited performing similar simulations under different conditions for the nonbond interactions considered in the used force field. This led to conclude that the width of this distribution is mainly controlled by the nonbond interactions. Moreover, these nonbond interactions also contribute significantly to the value of the average barrier for methyl group reorientation.

In a later work [69], the methyl group dynamics in PI was investigated well above the glass-transition temperature $T_{g}$. Starting from the self part of the van Hove correlation function, the incoherent intermediate scattering function was calculated for the protons in the main chain and in the methyl groups. The dynamics of the latter ones could be well described assuming decoupled segmental dynamics and rotations in a threefold potential. The distribution of potential barriers for methyl group rotation at such high temperature was found to be very similar to that deduced from low temperature MD-simulation results and inelastic neutron scattering measurements above mentioned [68]. Other works combining NS and simulations on poly(propylene oxide) (PPO) [53] and PMMA [70] well above the glass transition have also shown that the assumption of statistically independence for methyl group rotations and α-relaxation is a good approximation. The glass transition thus hardly modifies the energy landscape for this local process.

**Figure 5:**
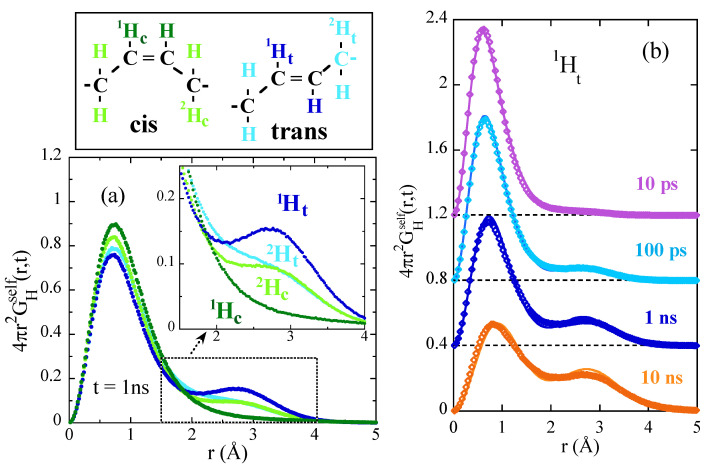
(**a**) Radial probability distribution function calculated at 200 K ($T_{g}$ + 20 K) and *t* = 1 ns for the different kinds of hydrogens in the 1,4-units of PB. The upper panel displays the schematic representation of the isomeric forms of 1,4-PB monomers and shows with colors the nomenclature for the different types of hydrogens. (**b**) Radial probability distribution function calculated for ${}^{1}$H${}_{t}$ (methyne hydrogen in the trans unit) at the same temperature at the different times indicated. For clarity the origins are shifted to the levels displayed by the horizontal dotted lines. The solid lines show the description obtained by the model proposed in Refs. [75,76].

Beyond methyl group dynamics, the rich microstructure of polymers—even in systems as ‘simple’ as PB—leads to a variety of dynamic processes active below as well as above their glass transition. These processes are behind the so-called secondary relaxations detectable by relaxation techniques like e.g., dielectric spectroscopy. In fact, for PB, there was a controversial situation regarding the origin of the dynamics observed by different techniques, mainly dielectric spectroscopy and NSE measurements close to the glass transition [71,72,73,74]. To disentangle the local motions of PB in this range, MD-simulations [75,76] of a realistic sample with similar microstructure as that of the real chains were carried out. The simulations were run for a very long time (≈1 μs!) to equilibrate the system as well as possible in the difficult temperature range under consideration (about 20 K above $T_{g}$). The results were carefully validated against NS data addressing structural as well as dynamical properties on both, protonated and deuterated samples [64,76]. Once the cell was validated, the radial probability distribution functions $G_{H}^{self}\left(r,t\right)$ corresponding to the different kinds of hydrogens in PB were calculated. Figure 5a shows that a second peak emerges in the distribution function, which is a are clear evidence for localized motions. Moreover, different hydrogens in the different units move in a rather different way. Having a look on longer times, contributions of diffusive kind (broadening of the distribution and shift to longer distances) can also be envisaged at this low temperature (see Figure 5b). These contributions become more dominant as the temperature increases. The underlying diffusive motion is the α-relaxation process we will address in the next section. Based on all these results, a simple model was constructed which combines hopping in an asymmetric double-well potential and an anomalous diffusion process representing the α-relaxation (see below) [75,76]. The model nicely fits the results in the real space, as can be seen in Figure 5b. Note that the assumption in the model to combine the motions was statistical independence of local and diffusive processes—the same as found for the simultaneous occurrence of methyl group rotations and segmental relaxation above commented. From the fits of the model, the hopping times of cis- and trans units were obtained. They turn out to be rather different. The reported dielectric results on the secondary β-relaxation are compatible with the timescales observed for the jumps involving the cis-units. This result is sensible, since the cis-unit is the moeity carrying the dipole moment of 1,4-PB. Conversely, the NSE values are rather close to those obtained for the local process of the trans-units. The short-time window accessed by NSE in the explored *Q*-range (below approx. 10 ns) is apparently more sensitive to the fastest motions occurring in the sample. Thus, MD-simulations allowed putting into a context the controversial puzzle. The intrinsic heterogeneous dynamics was the key question: cis- and trans-units behave in a rather different way and with different relaxation times.

#### 3.2.2. The Structural Relaxation

At the beginning of this century, through extensive NS investigations on protonated polymers, a universal behavior for the self-atomic (H) motions in the α-relaxation regime was established. Most experiments yielded $S_{H}^{inc}\left(Q,\omega \right)$ in the frequency domain. Roughly speaking, the width of the spectra is inversely proportional to the characteristic time of the process involved. This width depends on *Q*, what is a signature of an underlying diffusive-like process. However, contrarily to the case of simple diffusion, the results are not compatible with a simple exponential decay for the intermediate scattering function. In general, they can be accounted for by the Fourier Transform of a *stretched* exponential function for $I_{H}^{inc}\left(Q,t\right)$:

(4) $$I^{inc}\left(Q,t\right)=\exp\left[-{\left(\frac{t}{\tau_{w}}\right)}^{\beta }\right]$$

**Figure 6:**
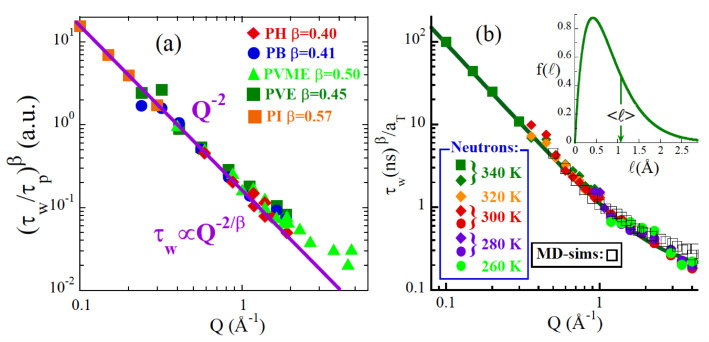
(**a**) Master curve giving the *Q*-dependence of $\tau_{w}$ constructed with results from different polymers (see legend; PH:phenoxy), applying polymer-dependent normalizing factors ($\tau_{p}$). The solid line is the Gaussian behavior. (**b**) Master curve built combining NS from different spectrometers and MD-simulations results for PId3 [85,86], applying *T*-dependent shift factors $a_{T}$. The solid line is the description by the anomalous jump diffusion model with the distribution of jump lengths in the inset. Adapted from [6] with permission from The Royal Society of Chemistry.

This function is characterized by the stretching exponent β<1 and a *Q*- and *T*-dependent characteristic time $\tau_{w}$ (see, e.g., [77]). This function is also commonly known as Kohlrausch-Williams-Watts (KWW). Furthermore, results on polymers reported over more than ten years [77,78] had shown that in the *Q*-regime approx. 0.2 $\leq Q\leq 1\;{Å}^{-1}$$\tau_{w}\left(Q\right)$ follows the law: $\tau_{w}\left(Q\right)\propto Q^{-2/\beta }$ (see e.g., Figure 6a). Considering this expression in Equation (4), one immediately arrives to the Gaussian *Q*-dependence of $I_{H}^{inc}\left(Q,t\right)$:

(5) $$I^{inc}\left(Q,t\right)=\exp\left[-\frac{\langle r^{2}\left(t\right)\rangle }{6}Q^{2}\right].$$

where the mean squared displacement of Hs in the α-relaxation increases *sublinearly* with time as $\langle r^{2}\left(t\right)\rangle \propto t^{\beta }$. This result qualifies the motions as an ’anomalous sublinear diffusion’. A perfect Gaussian behavior was in apparent contradiction to MD-simulations results on glass forming systems other than polymers (Lennard-Jones liquids [79,80], water [81], ortho-terphenyl [82] and Selenium [83,84]). Thus the question arose: Do glass forming *polymers* behave in a different way? Noteworthy, a close inspection to the high *Q*-data in Figure 6a already suggested deviations from the Gaussian law $\tau_{w}\left(Q\right)\propto Q^{-2/\beta }$.

At this point, a combination of fully atomistic MD-simulations [87] and NS measurements on the same polymer, PI, was used to clarify the situation [85,86]. Several spectrometers were used to extend as much as possible the investigated *Q*-range. NSE was particularly important. It allowed direct access to the deconvoluted intermediate scattering function in the time domain, for an accurate determination of its functional form. The sample investigated had deuterated methyl groups (PId3), such that the self motions of the main chain protons were the main contribution to the experimental results. These motions were also followed from the atomic trajectories delivered by the MD-simulations. Figure 6b shows the obtained *Q*-dependence of $\tau_{w}$ from the two methods. The results univocally confirm the Gaussian-like behavior in the *Q*-range approx. $Q\leq 1\;{Å}^{-1}$. At higher *Q*-values, clear signatures of deviations become evident.

The question then was the origin of the deviations observed. It was noted the strong reminiscence of the way the characteristic time departs from the Gaussian expectation (Figure 6) with the manifestation of the discrete nature of diffusion in simple jump diffusion models [88]. Basing on this similarity, a model was proposed, where a distribution of elementary jump lengths was assumed behind the anomalous diffusion carried out by the atoms in the α-process [85,86]. As shown in Figure 6b, such a simple approach provides a very good description of the *Q*-dependence of $\tau_{w}$ in the whole *Q*-range explored. The associated distribution of jump lengths f(ℓ) has a maximum at about 0.4 Å for PI (inset of Figure 6b). This suggests that a distribution of discrete step lengths is the origin of the universal deviations from Gaussian behavior in the self-motions of atoms during the structural relaxation regime. This model has also been successfully applied on other polymers in latter works [PB [89], poly(vinyl ethylene) (PVE) [89,90], poly(vinyl methyl ether) (PVME) [91], PMMA [70], poly(ethyl methacrylate) (PEMA) [92] and poly(tetrahydrofurane) (PTHF) [93]].

**Figure 7:**
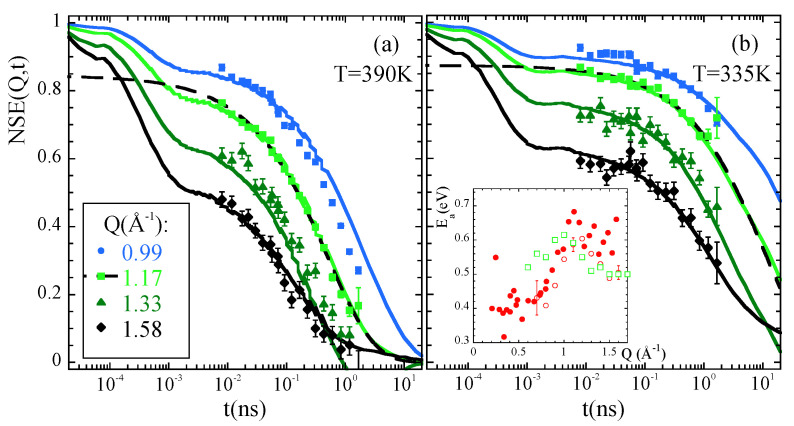
Comparison of the NSE results on a fully deuterated PIB sample [99] (symbols) and calculated from simulations [65] (solid lines) at 390 K (**a**) and 335 K (**b**) at the *Q*-values indicated in (**a**). Inset in (**b**) *Q*-dependence of the apparent activation energy for collective relaxation obtained from KWW fits of the experimental (solid) and simulated (empty) data. Examples of the fits are shown for $Q\approx Q_{max}$. Adapted with permission from [65]. Copyright (2014) American Chemical Society.

The information about H-self motions in the α-relaxation regime is of utmost interest in the field of glass-forming systems. However, in the framework of important theories for the glass transition as the Mode Coupling Theory (MCT) [2,94,95], the most relevant magnitude is the dynamic structure factor at the intermolecular correlations. This function is accessible by means of NS—in this case, on fully deuterated samples. The earliest experimental work by NSE on PB [96] was followed by others on PI [97], PIB [98,99], atactic polypropylene (a-PP) [100], polyurethene (PU) [101], poly(vinyl chloride) (PVC) [102], PVE [13], poly(ethylene propylene) (PEP) [103] PTHF [104]. In these works, the universality of the properties of the structural relaxation—stretching, scaling—was usually checked. The stretched character of the decay of the inter-molecular correlations (KWW-like functional form, Equation (4), see Figure 7) is nowadays a well established feature; however, the scaling property (time/temperature superposition principle) is not clearly always fulfilled (see, e.g., [103,104,105]).

**Figure 8:**
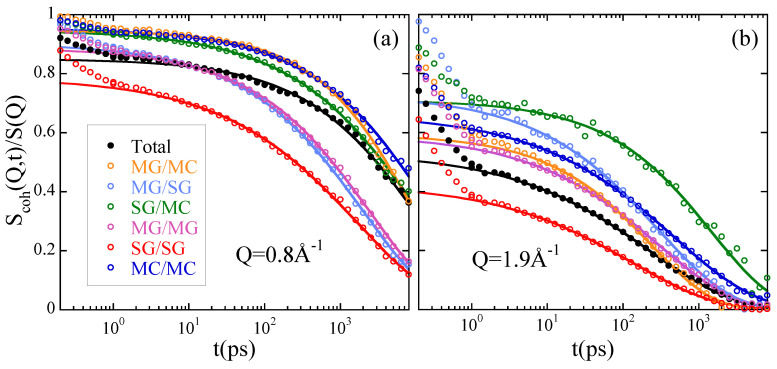
Normalized partial dynamic structure factors of deuterated PMMA corresponding to the different atomic groups considered [see the insert in (a) for the color code]. The total dynamic structure factor is shown in black. The lines are KWW descriptions for t≥4 ps. (**a**) $Q\;=\;0.8\;{Å}^{-1}$ (first structure factor peak); (**b**) $Q\;=\;1.9\;{Å}^{-1}$ (second structure factor peak). Adapted with permission from [70]. Copyright (2006) American Chemical Society.

Obviously the interpretation of collective motions is much more complicated than for the self-motions. Molecular Dynamics simulations have been used to complement experimental investigations on the dynamic structure factor in several cases [65,70,75,103]. Magnitudes that cannot be experimentally observed could be computed from the simulations, as they are the partial dynamic structure factors. An illustrative example is depicted for the case of deuterated PMMA in Figure 8 [70]. The observed temporal evolution of the total dynamic structure factor is the result of a complex interplay of the different partial contributions. At the first structure factor peak ($Q_{max}\;=\;0.8\;{Å}^{-1}$), the timescales for collective motion strongly depend on the molecular groups considered, and are spread over more than one order of magnitude. At the second peak ($Q_{2max}\;=\;1.9\;{Å}^{-1}$, basically all the relaxation times are within a factor of 2. The simulations also allow easily comparing collective and self-correlation functions, addressing the coherency of the relaxation process. For PMMA it was found that, while coherency effects are observed for all correlations at the first structure factor peak, at the second peak coherency remains only for correlations involving the main chains.

The combined consideration of NS and MD-simulations has also been of utmost help in exploring the applicability of theoretical frameworks, like the MCT, in polymers [106,107,108]. Consistent results were found when applying MCT to these systems, though with rather high values of the exponent parameter λ (the key parameter in this theory). The λ-values reported from other works for real polymers like PB [109], poly(propylene glycol) (PPG) [110] or PS [111], and simple polymer models *with* intramolecular barriers [112] are similarly high. Such finding could be interpreted in the framework of a higher-order MCT transition for real polymers arising from the simultaneous occurrence of two mechanisms leading to dynamic arrest: (i) packing, of intermolecular character and present in all glass-forming systems, and (ii) barriers for conformational changes, of intramolecular origin, which combined with chain-connectivity are specific of macromolecular systems [106,112].

#### 3.2.3. Chain Dynamics

At length scales much larger than the monomer size, polymer melts show unique dynamic processes that are controlled by chain connectivity and the size of the macromolecules. The rheological properties of polymer melts are ultimately determined by these processes. The so-called Rouse model [12] is the standard model to describe polymer chain dynamics in the melt. It considers the conformational entropy as the only source for restoring forces which stabilizes excursions from equilibrium. A coarse-grained model of *N* beads connected by entropic springs represents the chain, and a stochastic background gives account for the contribution of the surrounding chains. The created friction is characterized by the friction coefficient ξ. The Langevin equation that results from this situation can be solved by transforming to the normal coordinates (Rouse modes)

(6) $${\vec{X}}_{p}\left(t\right)=\frac{1}{N}\sum_{i=1}^{N}{\vec{R}}_{i}\left(t\right)\cos\left[\frac{p\pi }{N}\left(i-\frac{1}{2}\right)\right]$$

with $\vec{R_{i}}\left(t\right)$ the position of the *i*th bead of the *Gaussian* chain and *p* the mode number (p=0,1,2,3,⋯,N−1). In this model, the Rouse correlators $\langle \vec{X_{p}}\left(t\right)\vec{X_{p}}\left(0\right)\rangle$ relax independently and exponentially with *p*-dependent characteristic times. Conceptually, this model has two limitations. The first one arises at large distances. There, long chain melt topological constraints cause entanglements leading to the reptation mechanism. The other limitation is found at shorter distances. There, the assumptions allowing for a simplified coarse-grained chain cease to be valid. The local chain structure and dynamics become relevant, in particular approaching the intermolecular distances. As we have discussed above, there we approach the regime of the α process. The way how the crossover from segmental to Rouse dynamics takes place is still an intriguing question in polymer physics. To answer it, one of the key ingredients is to determine when the Rouse model ceases to be valid. This was the subject of a number of NS investigations on different polymers [113,114,115] in the past.

However, a thorough analysis of the sources for Rouse deviations is very difficult due to two factors: the uncertainties involved in the experiments on the one hand, and the impossibility to directly access the Rouse correlators, on the other hand. These problems are overcome in the simulations. With the current computing capabilities, big enough cells can be built to address with atomistic detail the chain dynamics in unentangled polymer systems.

A combined NS/MD-simulations strategy to tackle this problem has been applied to PB [116], PE [117], poly(ethylene oxide) (PEO) [118] and PEP [119]. After validation, magnitudes that cannot be accessed experimentally, like the mean squared displacement (msd) of different atoms or groups of atoms, were computed from the simulations, leading to noteworthy observations. For instance, inspection of the msd of the center of mass of the chains in PEP revealed a *sublinear* increase with time below $\tau_{R}$ (the longest relaxation time of the chain), presumably due to the effect of intermolecular interactions neglected in the Rouse model.

**Figure 9:**
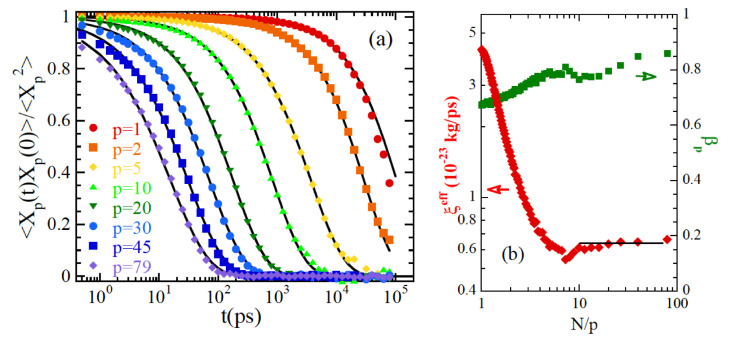
(**a**) Normalized Rouse correlators calculated for PEP at $T_{g}$ + 280 K and the mode-numbers indicated. Lines are fits with stretched exponentials. The obtained stretching parameters and effective friction coefficients (after ARS corrections) are displayed in (**b**). Rouse behavior corresponds to β = 1 and mode-independent friction (solid line). Adapted with permission from [119]. Copyright (2011) American Chemical Society.

In addition, by coarse graining the atomistic MD simulations a Rouse mode analysis of the simulated chains can be carried out. The results on the Rouse mode correlators are obtained defining a bead as the center of mass of a monomer. The such obtained correlators do not decay exponentially with time, but stretched exponential functions (like that in Equation (4)) have to be applied (Figure 9a). More pronounced stretching is observed when moving toward local length scales (small N/p-values) (Figure 9b). Moreover, at large *p*-numbers the *p*-dependence of the characteristic times strongly deviates from the Rouse prediction. An ARS-like approach [120] can account for the deviations originating from chain stiffness. However, these corrections are not sufficient to reproduce the results. The additional deviations can be characterized invoking of a *p*-dependent effective friction coefficient. This parameter decreases with the length scale, reaching a constant value (corresponding to the pure Rouse limit) at N/p≥8. The influence of the local potentials in the chain dynamics is clearly evidenced by these results. In the functions accessed experimentally by neutron scattering, these effects are reflected at high *Q* values and mainly short times.

Taking advantage of these well-validated atomistic MD-simulations of PEP, a bottom-up coarse-grained model was built based on them. With this model, the simulations could be extended beyond the entanglement mass of PEP [121]. Establishing the limits of chemically simplified models for direct mapping with experimental results is important. It is worth mentioning that these limits were thoroughly explored by other authors by comparing different levels of modeling for MD-simulations. They included explicit atom, united atom and coarse-grained simulations for two polymers, PEO [54] and PMMA [55].

Finally, we note that the Rouse scattering functions are based on the Gaussian approximation. The influence of non-Gaussian effects on the Rouse behavior was also studied by NS combined with atomistic MD-simulations in PB [48] and later in PEO forming part of an asymmetric polymer blend with PMMA [122].

## 4. Beyond ‘Simple’ Polymers and ‘Standard’ Experiments

In the following we present studies tackling even more intricate problems than those addressed in the previous section. A first step in the complexity of the polymers investigated can be given by considering still homopolymers, but with long or bulky side groups. This, as we will show next, gives rise to an interesting phenomenon: the nano-segregation of the chain components, that can be addressed by combining neutron scattering and MD-simulations. Thereafter, we will focus on a general problem of high relevance for glass-forming systems and liquids in general: the collective dynamics at intermediate length scales. We will show recent progresses in this almost white field, emphasizing novel results obtained by state-of-the art instrumentation that allows for polarization analysis on quasielastic experiments with high energy resolution.

### 4.1. Nanosegregation in More Complex Polymers

The structural and dynamical features of the family on poly(n-alkyl methacrylates) (PnMAs) and other polymers containing alkyl side groups were the focus of great interest in the past [58,123,124,125,126,127,128,129,130,131,132,133,134,135,136,137,138,139,140,141,142,143,144,145,146,147]. The main reason was the suggested formation of nano-domain structures by self-assembly of the side groups, that are segregated at nanometric scale from the main-chain subsytems. Such a morphology was deduced from the presence in the X-ray diffraction patterns of peaks at relatively low *Q*-values (’intermediate’-range), with side-group length dependent positions [134,138,146]. Neutron diffraction combined with isotopic labelling definitely proved such a scenario [58,143]. In addition, interesting peculiarities in the dynamics of the side groups were reported [58,138,143], in principle attributed to confinement effects induced by the surrounding main chains.

Later, the dynamics of the family of poly(alkylene oxides) (PAOs) were investigated by mechanical and dielectric spectroscopies [148] as well as by neutron scattering [149]. For these polymers, the side-groups motions did not show anomalies which could be attributed to confinement effects. If such effects emerged as a consequence of the nano-segregation of side groups and main chains, the question arose: Is the nano-segregation suppressed in PAOs by the large flexibility of the PEO-like main chain?

**Figure 10:**
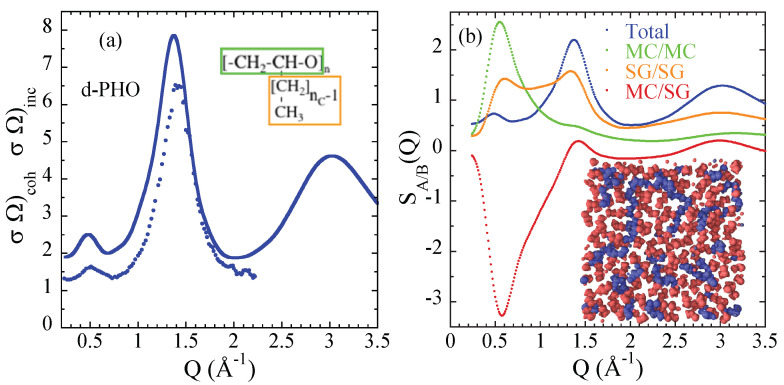
(**a**) Direct comparison of the ratio between coherent and incoherent differential cross sections measured at 300 K and calculated from the simulations at 360 K for fully deuterated PAO with $n_{C}$ = 4. (**b**) Contributions to the normalized total structure factors for this polymer calculated from the MD-simulations. This panel shows a slice of the simulation cell. Main-chain atoms are represented in blue, side-group atoms in red. The nano-segregation can be appreciated at first sight. The definitions of MC and SG subsystems are shown in the scheme of the monomer in (**a**) by the green and orange rectangles respectively. Adapted with permission from [150]. Copyright (2012) American Chemical Society.

A combined approach including neutron diffraction and MD-simulations allowed deciphering the structure of PAOs at short and intermediate (from Å to few nanometers) length scales [150]. Clear experimental evidences were found for the presence of alkyl nano-domains within which the local structure is even more similar to bulk PE than in the case of PnMAs. In addition, the properly validated simulated samples (with $n_{C}$= 2 and 4 alkyl carbons) provided direct proof of the nano-segregation and allowed calculating different magnitudes like the contributions of the main molecular groups (main chains and side groups) to the structure factor (see Figure 10 for the case of $n_{C}$ = 4). Thus, the lack of observation of confinement effects in the side-group dynamics of PAOs has to be attributed to other ingredients than the absence of structurally segregated phases. Large main chain flexibility leading to similar characteristic times for the dynamics of side groups and main chains could be invoked to rationalize the different dynamical behavior in PAOs with respect to PnMAs. Confinement effects in the latter would derive from the intrinsically different flexibility in main chains and side groups leading to a strong dynamic asymmetry. This effect is absent in PAOs. According to this scenario, the analysis of the dynamics from the simulations on the PAO with $n_{C}$ = 2 showed that for this polymer, full rotations of the pendant groups take place during the decaging of the main-chain segments [151].

It is worth noting that the universality of the emergence of nano-domains in polymers with long side groups was checked in a computational investigation of a simple bead-spring model for comb-like polymers [152]. Removing the particular chemical details, the systems constructed mimicked families of polymers like PAOs or PnMAs. That study showed that nanosegregation of main chains and side groups can arise as a purely entropic effect, provided that the density of branch points is large enough.

Another polymer with relatively complex composition, poly(vinylpyrrolidone) (PVP), was also investigated by combining NS, X-ray diffraction, and fully atomistic molecular dynamics simulations [153]. A “prepeak” in the X-ray diffraction pattern shows the typical features of a first amorphous halo. The experimentally accessed incoherent scattering function of hydrogens exhibited enhanced stretching and a *Q*-dependence of the characteristic time different from that usually reported for simpler polymers (main-chain polymers or polymers with small side groups). The comparison with both kinds of experimental results validated the simulations. Nanosegregation of side groups and main chains was suggested by the analysis of the simulated structure factor. Furthermore, the detailed insight provided by the simulations on the atomic trajectories revealed a partial and spatially localized decoupling of MC and SG dynamics at length scales between the average SG–SG distance and the characteristic length of the backbone interchain correlations. Correlators calculated for the SG subsystem showed anomalous behavior, like e.g., logarithmic-like decays of the density–density correlation function. They might be again a consequence of the existing large dynamic asymmetry between SG and MC subsystems. It was proposed that, as the SGs are spatially extended and chemically different from the backbone, they form transient nanosegregated domains. The dynamics of these domains showed similar behavior to that found in other systems displaying large dynamic asymmetry. We note that this ingredient has been found to be also crucial in e.g., polymer blends [154].

### 4.2. The Collective Dynamics at Intermediate Length Scales: An Unknown Territory

A particularly interesting region, not only for polymers, but for glass-forming systems and liquids in general, is the so-called intermediate length scales (ILS) region—the range of length scales larger than the intermolecular distances but not yet in the hydrodynamic regime [65,155,156]. In polymers, this is also the region where the α-relaxation crosses over toward the Rouse dynamics. This problem has been addressed above (Section 3.2.3) for the single chain dynamic structure factor. However, the general question how collective dynamics develop in this regime—in liquids and melts—is poorly understood. In fact, from a theoretical point of view, the connection between collective and self-motions is an unsolved problem in complex systems. The relationship proposed by deGennes [157], that the collective time is just the product of the self-correlation time and the structure factor, $\tau_{coh}\left(Q\right)=S\left(Q\right)\tau_{s}\left(Q\right)$, would be strictly applicable only to monoatomic liquids in the range of the first maximum of S(Q). In the case of anomalous diffusion, where stretched exponentials are found instead of single exponentials, the ansatz proposed by Sköld [158] relating the intermediate scattering functions $I_{coh}\left(Q,t\right)=I_{s}\left(Q/\sqrt{S(Q)},t\right)$ might be applied. This translates in a modified version of the deGennes narrowing as $\tau_{coh}\left(Q\right)=S{\left(Q\right)}^{1/\beta }\tau_{s}\left(Q\right)$. From an experimental point of view, for polymers, this question has been addressed first only by NS [99,159], and, later, by the combination of NS and MD-Simulations [65,103,160]. Though the Sköld-like ansatz seems to work reasonably well in some cases close to the first structure factor peak, deviations are found away from this inter-molecular correlations region. For instance, in the case of PIB, a marked *Q*-dependence of the apparent activation energy was found for coherent scattering (see inset in Figure 7b) [65,99] rendering any ‘simple’ connection with incoherent scattering as inviable, even more at ILS.

Combining NS and MD-simulations it was possible to describe in a rather satisfactory way the collective behavior of PIB not only close to the first structure factor peak but also approaching the ILS. The starting point was the ansatz proposed in Ref. [111], where the collective time is given by

(7) $$\frac{1}{\tau (Q)}=\frac{1}{\tau (Q\to 0)}e^{-Q^{2}\xi_{c}^{2}}+\frac{1}{\tau^{D}\left(Q\right)}.$$

**Figure 11:**
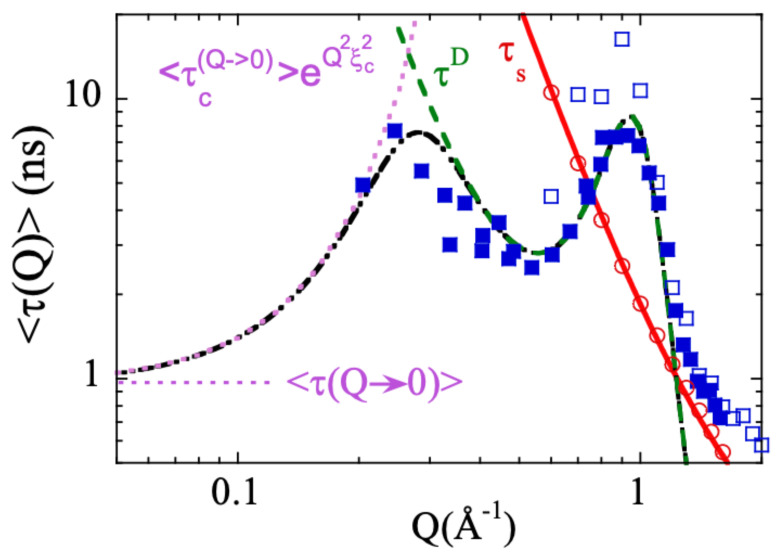
*Q*-dependence of the average characteristic time for collective (empty squares) and self-correlation (circles) functions of PIB obtained from simulations. Filled squares are the experimental collective times [99]. Solid line: description of the self-correlation times by the anomalous jump diffusion model. Dashed-dotted line: description of the collective times by the proposed model [65,155,156]. The dotted line represents the diffusive contribution and the dashed line the Q→0 contribution, with the location of the non-diffusive time indicated by the horizontal line. Adapted with permission from [65]. Copyright (2014) American Chemical Society.

The non-diffusive (*Q*-independent) time τ(Q→0) should reflect the viscoelastic coupling of stress and density fluctuations on scales long enough compared to atomic dimensions, but not yet in the hydrodynamic limit [161]. Its contribution to the total collective time is affected by a Gaussian cutoff factor $e^{-Q^{2}\xi_{c}^{2}}$ to ensure that it is present only on length scales beyond a characteristic length $\xi_{c}$, which is assumed to be $\xi_{c}∼2\pi /Q_{I}$. The diffusive time $\tau_{D}$ was generalized by us to the case of anomalous diffusion as above described by the Sköld approximation. To apply the model to PIB, we used the information on the total self-motions available from MD-simulations properly validated by direct comparison with experimental results. The collective time at Q→0 was extracted from the fits of the coherent NSE data. This time agrees very well with compiled results from different experimental techniques directly accessing such relaxation time. Figure 11 shows the different components involved in the model. This generalized model also gives account for the modulation of the apparent activation energy of the collective times with the static structure factor (inset of Figure 7b). It would mainly result from changes of the short-range order at inter-molecular length scales [65,156]. This model also allows a semiquantitative description of recent experimental results on PTHF [93,104].

Despite these advances, the ILS region is still hardly explored in glass-forming systems and liquids. A severe complication for the experiments derives from the difficulties in separating coherent and incoherent contributions from the measured signal. Though the coherent scattering cross section is much higher than the incoherent one in fully deuterated systems, the small amplitude of the density fluctuations away from the first amorphous halo, in particular at low-*Q* values, leads to very weak signals, comparable there to the incoherent contributions. The parasitic incoherent contribution is largely (but not completely) suppressed by the NSE technique [99]. As mentioned above, currently, polarization analysis (PA) allowing for a full separation is not routinely implemented in neutron spectrometers due to a series of technical difficulties [162,163]. For this reason, extending the experimental investigations toward the ILS is a real challenge.

### 4.3. A Breakthrough Experiment on Water: A ‘Simple’ and Yet Enormously Complex System

**Figure 12:**
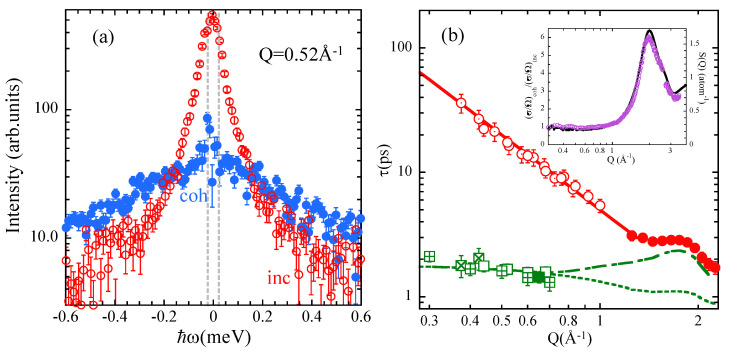
(**a**) Comparison of the coherent and incoherent dynamical structure factors measured by LET on D${}_{2}$O at 295 K at $Q\;=\;0.52\;{Å}^{-1}$ [19]. Vertical dotted lines represent the resolution FWHM. (**b**) *Q*-dependence of the characteristic relaxation times: $\tau_{d}$ (circles) and $\tau_{c}$ (squares; from MD-simulations, squares with crosses). The $\tau_{d}$ values have been obtained from incoherent scattering ($\tau_{d}^{inc}$) and from the fit of the coherent scattering results to the model (•). Solid line: fit of $D^{-1}Q^{-2}$ to $\tau_{d}^{inc}$. $\tau_{c}$ and 〈τ〉-values calculated with the model are shown by the dashed and dashed-dotted lines respectively. The temperature is 295 K [19]. Inset: Ratio between coherent and incoherent differential cross sections of D${}_{2}$O at 298 K (hollow dots) as function of *Q* and calculated from MD-simulations (solid line). Adapted from [19].

Recently, a few multi-detector spectrometers have demonstrated the capability of performing neutron spectroscopy with PA and sub-meV resolution [164,165,166]. We took this unique opportunity to scrutinize the coherent structural relaxation in a wide *Q*-range from meso- to inter-molecular scales in the most relevant liquid: water [19]. We note the relevance of water also in different polymer and bio-polymer systems. The experiments were carried out on the LET direct geometry time-of-flight spectrometer at the ISIS Neutron and Muon Source, Oxfordshire, UK. Figure 12a shows the coherent and incoherent contributions to the total spectrum, for a *Q*-value in the ILS region (see structure factor of water in the inset of Figure 12b). The quality of the results is absolutely amazing, considering the extremely low coherent signal in this regime. As can be appreciated just at first sight, coherent and incoherent scattering are extremely different. The coherent contribution is very broad in comparison with the incoherent one. As this broadening is a measure of the inverse of the corresponding relaxation time (FWHM ∼ 1/τ), this tells us—without any fitting—that the coherent characteristic time $\tau_{c}$ is of the order of 10 times smaller than the incoherent (diffusive) relaxation time. The latter is close to that deduced from the total (incoherent plus coherent) spectra that is measured if PA is not used. Since the relaxation time at Q≈ 0.5 Å${}^{-1}$ reported from measurements on D${}_{2}$O at 298 K without PA is ≈20 ps [167], then $\tau_{c}$ should be ≈2 ps.

In the low-*Q* meso-scale, the observed decorrelation of the collective fluctuations hardly depended on *Q*. These results were also corroborated by MD-simulations on a big cell (see Figure 12b). In the crossover range towards inter-molecular scales—where the structural relaxation is dominated by diffusion–, the experimental results were nicely described by the convolution of a *Q*-independent mode and diffusion. The model applied is very general, and had been used previously to describe the merging of the α and β processes in glass-forming polymers [71,72,168] and more recently to analyze the incoherent NS and NMR results of water [167,169]. We address the interested reader to Ref. [19] for more details about this work.

## 5. Conclusions

We have presented a general and robust methodology consisting of the synergetic combination of neutron scattering experiments on real samples and fully atomistic MD-simulations, to face challenges in diverse areas, including in particular the field of polymer melts and glass-forming polymers. The success of the methodology here presented has been illustrated with examples covering a wide range of systems of diverse complexity and problems of different nature. We have shown the added value of the combination of neutron scattering and fully atomistic MD-simulations in deciphering the structural properties of chemically simple polymers as well as of other systems with long or bulky side-groups. The potential of this strategy has also been highlighted in the case of the dynamical studies; we have shown a substantial increase in the understanding of different processes characteristic for polymers, ranging from the simplest motions (methyl group rotations) to the Rouse dynamics, passing through the local processes involved in the secondary relaxations and the structural relaxation. The power and versatility of the tandem composed of neutron scattering and fully atomistic MD-simulations has thus largely been demonstrated along these years of application by our group.

Many interesting questions in the field of polymers, however, remain of course open and are still challenging. A large number of them relate to multi-component systems and/or to systems containing macromolecules with complex architectures or displaying relevant features at mesoscopic scales. These problems are in general not affordable by the combination here presented. Still in these cases, neutron scattering on properly H/D labeled systems can provide the right experimental tool to scrutinize such systems. However, the design of the adequate samples is usually very delicate and in general demands for advanced chemical synthesis capabilities involving deuteration. The experiments involve then small angle neutron scattering (SANS) and NSE techniques at low *Q*-values, addressing the static and dynamical features at the mesoscale, and the interpretation of the results can also be very complicated. Support of simulations becomes then also extremely useful. But in such cases, coarse-grained simulations are the good choice. Forgetting about the chemical details, they can be used to explore generic features and/or very big systems. In this review, when presenting the PAOs studies, we have already mentioned the interesting result of coarse-grained investigations on comb-like polymers addressing the generality of the nano-segregation effect [152,170]. Thus, the methodology here proposed can be extended to this kind of investigations but using coarse-grained models instead of fully atomistic simulations. Another case where this synergy has recently been exploited is in the field of single-chain nano-particles (SCNPs) [171,172,173,174,175]. These unimolecular nano-objects obtained by intramolecular cross-linking of individual macromolecular chains are highly promising in the field of nanotechnology. Combining these two tools—SANS and/or NSE on H/D labelled samples and coarse-grained simulations–, deep insight into the fractal dimension and the internal friction characterizing these fascinating macromolecules has been gained.

Special mention deserve, concerning the use of coarse-grained models, the studies where the microstructure of the polymer plays a relevant role, even though the focus is on relatively large length scales as compared with the intermolecular distances. This could be for example the case of random copolymers. In such works, fully atomistic simulations can be of utmost help providing characteristic parameters of polymer chains as, for instance, the statistical segment length. We have already mentioned the case of PEP, where atomistic MD-simulations validated with experiments were used to build a bottom-up coarse-grained model to extend the simulations beyond the entanglement mass [121].

Finally, we have also shown that to tackle some intriguing problems, new experimental developments are crucial, as, for instance, the implementation of polarization analysis in quasielastic neutron scattering instruments. In fact, our recent work on D${}_{2}$O [19] has opened a new way of approaching the unknown territory of coherent scattering—from meso- to inter-molecular scales—not only in water under different conditions but also in H-bonded liquids, glass-forming liquids and biological systems where water plays an important role. It also convincingly proved the power of these capabilities, that can hugely impact the progress of microscopic dynamics investigations in fields like soft matter or biology.

## Figures and Tables

**Table 1:** Values of the average scattering lengths $\bar{b_{\alpha }}$, their squares ${\bar{b_{\alpha }}}^{2}$ and their deviations $\bar{\Delta b_{\alpha }^{2}}$, as well as the coherent and incoherent scattering cross sections for different isotopes α (1 barn = 100 fm${}^{2}$).

Isotope α	$\bar{b_{\alpha }}$/fm	${\bar{b_{\alpha }}}^{2}$/fm${}^{2}$	$\bar{\Delta b_{\alpha }^{2}}$/fm${}^{2}$	$\sigma_{\mathrm{coh}}^{\alpha }$/barns	$\sigma_{\mathrm{inc}}^{\alpha }$/barns
${}^{1}$H	−3.7406	13.992	638.78	1.7583	80.26
${}^{2}$H (D)	6.6710	44.502	16.322	5.592	2.05
${}^{12}$C	6.6511	44.237	0	5.559	0
${}^{16}$O	5.8030	33.675	0	4.232	0

## Abbreviations

The following abbreviations are used in this manuscript:

NS	Neutron scattering
MD	Molecular dynamics
NSE	Neutron spin echo
PA	Polarization analysis
PE	Polyethylene
AMBER	Assisted Model Building with Energy Refinement
COMPASS	Condensed-phase Optimized Molecular Potentials for Atomistic Simulation Studies
CHARMM	Chemistry at Harvard Macromolecular Mechanics
GROMOS	Groningen Molecular Simulation
OPLS	Optimized Potentials for Liquid Simulations
NAMD	Nanoscale Molecular Dynamics
GAFF	Generalized AMBER Force Field
GROMACS	Groningen Machine for Chemical Simulations
LAMMPS	Large-scale Atomic/Molecular Massively Parallel Simulator
OCTA	Open Computational Tool for Advanced material technology
PMMA	Poly(methyl metacrylate)
MC	Main chain
MG	Methyl group
SG	Side group
PI	Polyisoprene
PS	Polystyrene
PB	1,4-Polybutadiene
PIB	Polyisobutylene
RRDM	Rotational rate distribution model
PVAc	Poly(vinyl acetate)
PPO	poly(propylene oxide)
KWW	Kohlrausch-Williams-Watts
PId3	Polyisoprene with deuterated methyl groups
PVE	Poly(vinyl ethylene)
PVME	poly(vinyl methyl ether)
PEMA	Poly(ethyl methacrylate)
PTHF	Poly(tetrahydrofurane)
a-PP	atactic Polypropylene
PU	Polyurethene
PVC	Poly(vinyl chloride)
PEP	Poly(ethylene propylene)
MCT	Mode Coupling Theory
PPG	Poly(propylene glycol)
ARS	All rotational state model
PEO	Poly(ethylene oxide)
msd	mean squared displacement
PnMAs	Poly(n-alkyl methacrylates)
PAOs	Poly(alkylene oxides)
PVP	Poly(vinylpyrrolidone)
ILS	Intermediate length scales
NMR	Nuclear magnetic resonance
SANS	Small angle neutron scattering
SCNPs	Single-chain nano-particles
